# Supplementary material for: Isogenic Mammary Models of Intraductal Carcinoma Reveal Progression to Invasiveness in the Absence of a Non-Obligatory In Situ Stage
Source: Cancers (Basel). 2023 Apr 12;15(8):2257. doi: 10.3390/cancers15082257 (PMC10136757; doi:10.3390/cancers15082257)
Supplement: Supplementary file 1 [file cancers-15-02257-s001.zip › cancers-2311154-supplementary.pdf]

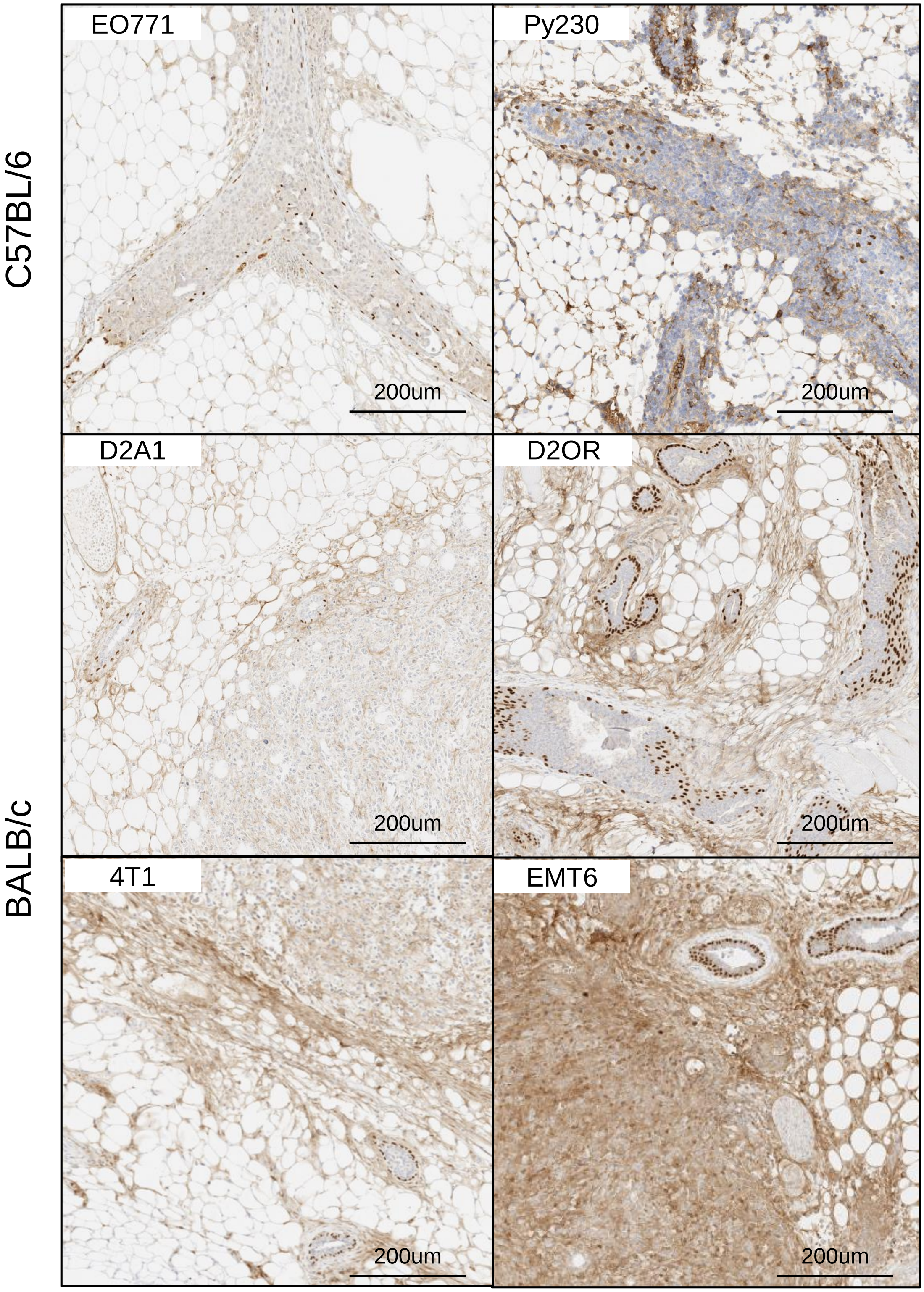

Supplementary Figure S1: p63 staining in intraductal tumors collected from C57BL/6 (EO771, Py230) and BALB/c (D2A1, D2OR, 4T1, EMT6) mice.

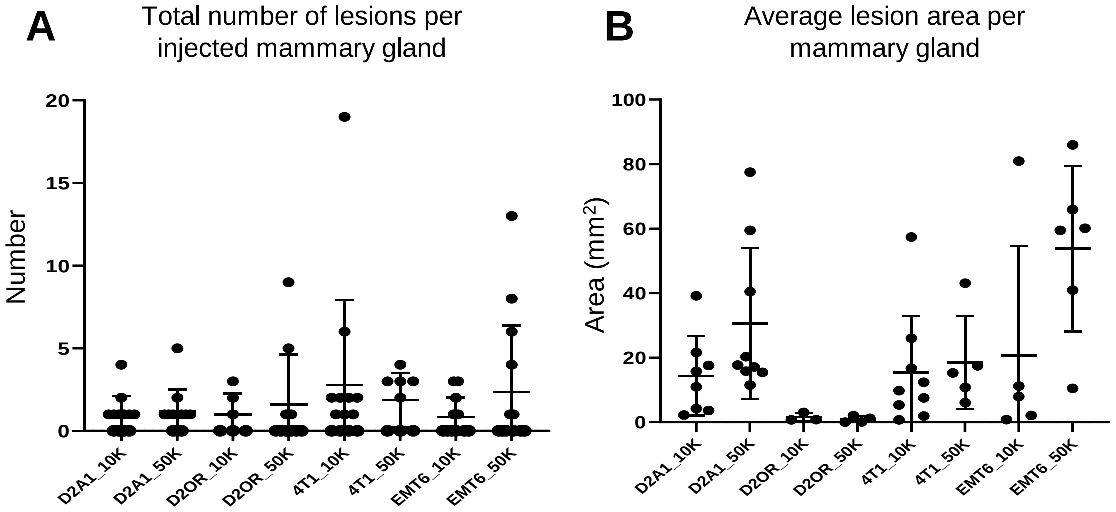

Supplementary Figure S2: The total number and average area of lesions that developed following intraductal injection of mammary cell lines into BALB/c immune-competent hosts. Murine mammary cell lines D2A1, D2.OR, 4T1, and EMT6 were intraductally injected into the 4th mammary glands of BALB/c mice at either or 1×10^4 (10K) or 5×10^4 (50K) cells in 2µL. Mammary glands were collected when the first tumor became palpable. (A) The average number of lesions per mammary gland. (B) The average area of lesions. Results presented as mean±stdev. Dots represent individual mice. Differences in tumor number and area were assessed using Student's T-test. No data were statistically significant.

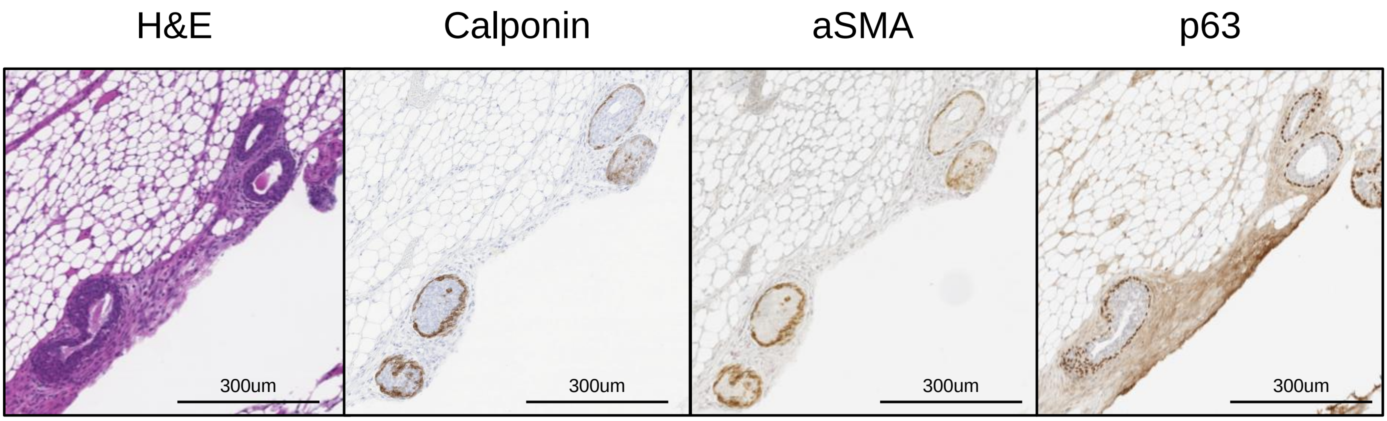

Supplementary Figure S3: Examples of DCIS collected from BALB/c (4T1) mice.

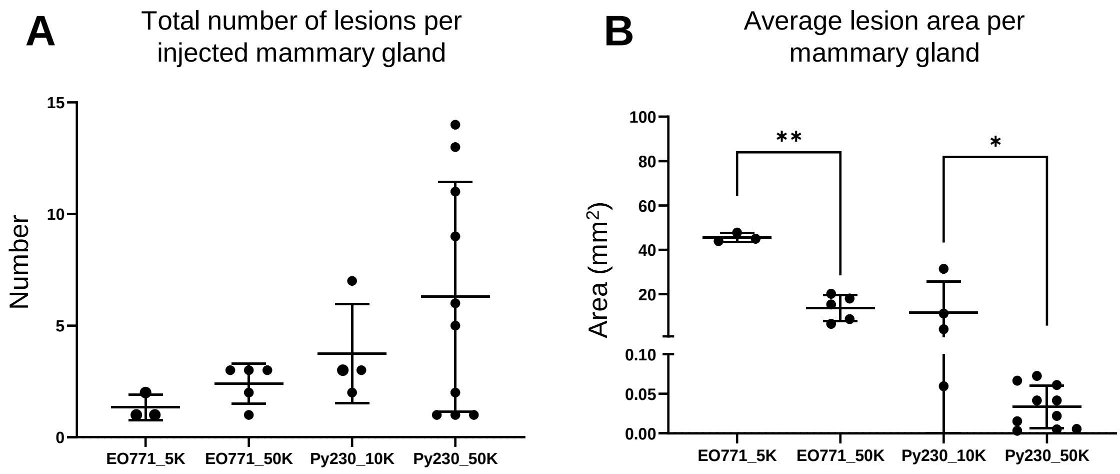

Supplementary Figure S4: The total number and average area of lesions that developed following intraductal injection of mammary cell lines into C57BL/6 immune-competent hosts. Murine mammary cancer cell lines EO771 and Py230 were intraductally injected into the 4th mammary glands of C57BL/6 mice at various concentrations: EO771 (5×10^3 (5K) or 5×10^4 (50K) cells in 2µL) or Py230 (1×10^4 (10K) or 5×10^4 (50K) cells in 2µL). Mammary glands were collected either 35 days (EO771) or 14 days (Py230) post-injection and assessed for tumors. (A) The average number of lesions per mammary gland. (B) The average area of lesions. Results presented as mean±stdev. Dots represent individual mice. Differences in tumor number and area were assessed using Student's T-test. $^{**}p=<0.005$; $^{*}p<0.05$

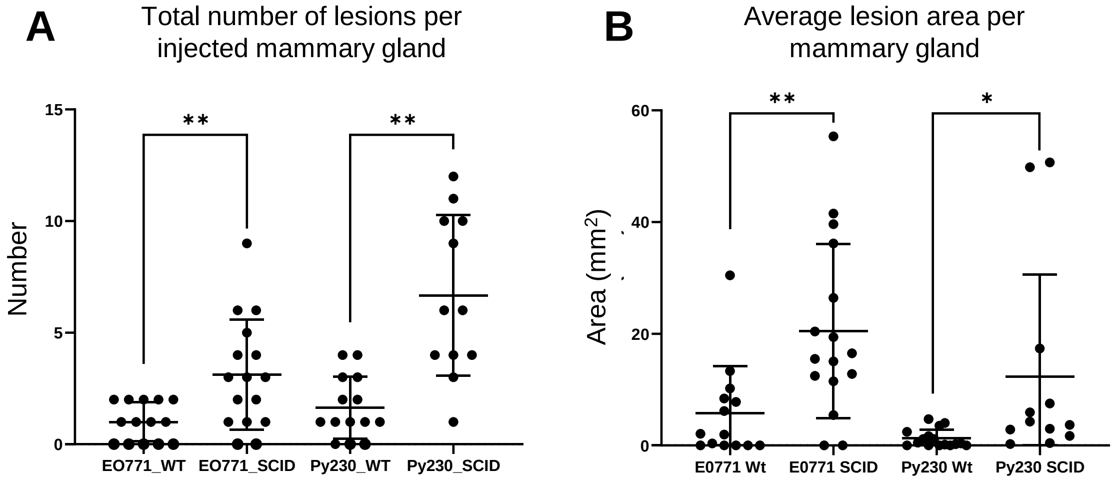

Supplementary Figure S5: The total number and average area of lesions that developed following intraductal injection of mammary cell lines into C57BL/6/SCID immunocompromised hosts. Murine mammary cell lines EO771 (5×10^4 cells in 2µL) or Py230 (5×10^4 cells in 2µL) were intraductally injected into the 4th right mammary glands of C57BL/6/SCID (SCID) mice or wild-type C57Bl/6 (WT) mice. Mammary glands were collected 26 days (EO771) or 11 days (Py230) later and assessed for tumors. (A) The average number of lesions per mammary gland. (B) The average area of lesions. Results presented as mean±stdev. Dots represent individual mice. Differences in tumor number and area were assessed using Student's T-test. **p=<0.005; *p<0.05

Cell line	Strain	Intrinsic Subtype	Ref.
D2A1	BALB/c	Luminal B	(Yang *et. al*., 2017)
D2OR	BALB/c	Basal-like	(Prunier *et. al*., 2021)
4T1	BALB/c	Luminal A	(Yang *et. al*., 2017)
EMT6	BALB/c	Luminal A	(Yang *et. al*., 2017)
EO771	C57BL/6	Luminal B	(Yang *et. al*., 2017)
Py230	C57BL/6	Luminal A, Her2+, Basal-like	(Bao *et al.,* 2015)

Supplementary Table S1: Intrinsic subtypes of the murine mammary cancer cell lines used in the study.

		MAMMARY GLAND				LESIONS				
	**Cell line**	**MG injected**	**Injections resulting in DCIS**	**Injections resulting in IDC**	**Injections resulting in no lesions**	**Total lesions across**	**Total DCIS lesions**	**Total IDC lesions**	**Average lesion size (mm^2)**	**Days to euthanasia**
**C57BL/6**	EO771 5K	12	0/12 (0)	3/12 (25)	9/12 (75)	4	0/4 (0)	4/4 (100)	45.58	35
	EO771 50K	14	0/14 (0)	5/14 (36)	9/14 (64)	12	0/12 (0)	12/12 (100)	17.78	35
	Py230 10K	5	0/5 (0)	4/5 (80)	1/5 (20)	15	0/15 (0)	15/15 (100)	11.74	14
	Py230 50K	11	1/11† (9)	10/11 (91)	1/11 (9)	63	6/63 (10)	57/63 (90)	0.322	14
**BALB/c**	D2A1 10K	12	0/12 (0)	8/12 (67)	4/12 (33)	12	0/12 (0)	12/12 (100)	14.40	33
	D2A1 50K	12	0/12 (0)	9/12 (75)	3/12 (25)	14	0/14 (0)	14/14 (100)	30.59	33
	D2OR 10K	6	0/6 (0)	3/6 (50)	3/6 (50)	6	0/6 (0)	6/6 (100)	1.55	15
	D2OR 50K	10	0/10 (0)	4/10 (40)	6/10 (60)	16	0/16 (0)	16/16 (100)	0.86	15
	4T1 10K	10	1/10† (10)	9/10 (90)	1/10 (10)	36	3/36 (8)	33/36 (92)	15.32	11
	4T1 50K	5	0/5 (0)	5/5 (100)	0/5 (0)	15	0/15 (0)	15/15 (100)	18.54	11
	EMT6 10K	11	1/11† (9)	5/11 (45)	6/11 (55)	10	1/10 (10)	9/10 (90)	20.58	14
	EMT6 50K	8	0/8 (0)	6/8 (75)	2/8 (25)	33	0/33 (0)	33/33 (100)	53.81	14

Supplementary Table S2: Summary of the number of ductal carcinoma *in situ* and invasive ductal carcinoma lesions that developed following intraductal injection of mammary carcinoma cell lines into immune-competent mice. Murine mammary cancer cell lines were intraductally injected into the 4th mammary glands of either C57BL/6 (cell lines EO771, Py230) or BALB/c (cell lines D2A1, D2OR, 4T1, EMT6) mice. Cells were delivered in a total volume of 2µL per mammary gland. Various cell concentrations were investigated, including 5×10^3 cells (5K), 1×10^4 cells (10K), or 5×10^4 cells (50K). Intraductal injections that resulted in both DCIS and IDC lesions in the same mammary gland are identified by †. Abbreviations MG = mammary gland; IDC = invasive ductal carcinoma; DCIS = ductal carcinoma *in situ*.

		MAMMARY GLAND				LESIONS				
	**Cell line**	**MG injected**	**Injections resulting in DCIS**	**Injections resulting in IDC**	**Injections resulting in no lesions**	**Total lesions across**	**Total DCIS lesions**	**Total IDC lesions**	**Average lesion size (mm^2)**	**Days to euthanasia**
**C57BL/6**	EO771 WT	14	0/14 (0)	9/14 (64)	5/14 (36)	9	0/9 (0)	9/9 (100)	5.77	26
	EO771 SCID	16	0/16 (0)	14/16 (88)	2/16 (12)	64	0/64 (0)	64/64 (100)	20.51	26
	Py230 WT	14	1/14[†] (7)	11/14 (79)	3/14 (21)	23	3/23 (13)	20/23 (87)	1.25	11
	Py230 SCID	12	0/12 (0)	12/12 (100)	0/12 (0)	80	0/80 (0)	80/80 (100)	12.29	11

Supplementary Table S3: Summary of the number of ductal carcinoma *in situ* and invasive ductal carcinoma lesions following intraductal injection of mammary carcinoma cell lines into immunocompromised mice. Murine mammary cell lines EO771 (5×10^4 cells in 2µL) or Py230 (5×10^4 cells in 2µL) were intraductally injected into the 4th right mammary glands of C57BL/6/SCID (SCID) mice or wild-type C57Bl/6 (WT) mice. Mammary glands were collected 26 days (EO771) or 11 days (Py230) later and assessed for tumors. Intraductal injections that resulted in both DCIS and IDC lesions in the same mammary gland are identified by †. Abbreviations MG = mammary gland; IDC = invasive ductal carcinoma; DCIS = Ductal Carcinoma *in situ*.
